# Pyrophosphate Regulates Multilineage Differentiation in Stem Cells From Human Exfoliated Deciduous Teeth

**DOI:** 10.1002/cre2.70248

**Published:** 2025-11-20

**Authors:** Ravipha Suwittayarak, Nunthawan Nowwarote, Chatvadee Kornsuthisopon, Waleerat Sukarawan, Brian L. Foster, Hiroshi Egusa, Thanaphum Osathanon

**Affiliations:** ^1^ Center of Excellence for Dental Stem Cell Biology, Faculty of Dentistry Chulalongkorn University Bangkok Thailand; ^2^ Department of Oral Biology, Faculty of Dentistry Université Paris Cité Paris France; ^3^ Department of Anatomy, Faculty of Dentistry Chulalongkorn University Bangkok Thailand; ^4^ Department of Paediatric Dentistry, Faculty of Dentistry Chulalongkorn University Bangkok Thailand; ^5^ Division of Biosciences, College of Dentistry The Ohio State University Columbus Ohio USA; ^6^ Division of Molecular and Regenerative Prosthodontics Tohoku University Graduate School of Dentistry Sendai Miyagi Japan

**Keywords:** bone remodelling, differentiation, pyrophosphate, sequencing, tooth

## Abstract

**Background:**

To explore the cellular behavior of stem cells derived from human exfoliated deciduous teeth (SHED) in response to inorganic pyrophosphate (PP_i_).

**Materials and Methods:**

SHED cells were isolated from the dental pulp tissues of human primary exfoliated teeth. Cell proliferation was examined using the MTT assay, colony‐forming unit assay, and cell cycle analysis. Cell migration was evaluated using the scratch assay. Osteogenic differentiation was assessed by the expression of osteogenic marker genes and *in vitro* mineral deposition. Oil Red O staining was employed to determine intracellular lipid accumulation under adipogenic differentiation. For osteoclast differentiation, TRAP staining was used. The global gene expression profile was examined by RNA sequencing analysis.

**Results:**

PP_i_ reduced early cell apoptosis and enhanced cell migration. PP_i_ inhibited mineral deposition dose‐dependently and significantly reduced *DSPP* and *BGLAP* expression. The higher dose of 10 μM PP_i_ decreased *RANKL* mRNA expression, while it did not influence *OPG* mRNA levels, resulting in the reduction of the *RANKL/OPG* expression ratio. Culture medium from PP_i_‐treated SHED reduced the number of TRAP‐positive multinucleated cells. Further, PP_i_ inhibited *CEBPA* but not *PPARG* and *LPL* mRNA expression under adipogenic induction. The intracellular lipid accumulation tended to decrease in PP_i_‐treated conditions (10 μM). The transcriptomic profiles illustrated that PP_i_ potentially modulated several pathways, including the metabolism of lipids, interleukin‐6, TGF‐β1, and NOTCH signaling.

**Conclusion:**

PP_i_ inhibited osteo/odontogenic, adipogenic and indirectly attenuated osteoclast differentiation by SHED. This study implicated that PP_i_ can modulate the cellular responses of SHED.

## 1 Introduction

Inorganic pyrophosphate (PP_i_) is composed of two inorganic phosphate (P_i_) ions linked with an ester bond. PP_i_ is a potent regulator of mineralization at micromolar concentrations, inhibiting hydroxyapatite (HA) growth (Foster et al. 2012; Orriss et al. 2016; Russell et al. 1971a). Whether mineralization is favored is thought to depend on the balance between concentrations of inorganic phosphate (P_i_), a component of HA, and PP_i_ concentration, depending on the stage of mineralization (Thouverey et al. 2009; Harmey et al. 2004).

Levels of PP_i_ are controlled by several regulatory proteins, including progressive ankylosis protein (ANKH), ectonucleotide pyrophosphatase phosphodiesterase 1 (ENPP1), and tissue‐nonspecific alkaline phosphatase (TNAP) (Harmey et al. 2004; Zhou et al. 2012). Two of these regulators are responsible for increasing extracellular PP_i_ levels. Transmembrane protein ANKH regulates the export of intracellular PP_i_ and/or adenosine triphosphate (ATP) (Ho et al. 2000; Szeri et al. 2020). Ectoenzyme ENPP1 hydrolyzes purine nucleotides like ATP to increase extracellular PP_i_ levels (Harmey et al. 2004). PP_i_ is a physiological substrate of TNAP, which hydrolyzes PP_i_ to release P_i_ ions for promoting biomineralization (Harmey et al. 2004; Millán and Whyte 2016). Loss‐of‐function variants in each of them cause improper mineralization, resulting in pathological conditions (Harmey et al. 2004; Foster et al. 2024). Effects of altered PP_i_ levels on mineralization in these conditions have been described; however, effects on other cell functions remain largely unexplored and unappreciated as a contributory mechanism.

There is growing evidence that PP_i_ can regulate cell fate determination, including osteogenic potential. PP_i_ induced early osteoblastic differentiation in mesenchymal stem cells (MSC), MC3T3‐E1 murine pre‐osteoblasts, and SaOS‐2 osteoblast‐like cells, demonstrating effects on cells over a wide range of differentiation potential (Pujari‐Palmer et al. 2016; Svensson et al. 2022). Higher PP_i_ concentrations sustained cell survival of mesenchymal stem cells (MSC) and fibroblasts (Svensson et al. 2022; Rubin and Bowen‐Pope 1979). The role of PP_i_ in directing other lineage determination has also been indicated, including chondrogenic differentiation (Johnson et al. 2005). Direct PP_i_ exposure to mesenchymal stem cells resulted in reduced expression of the chondrogenic marker gene, *SOX9* (Svensson et al. 2022 *)*. PP_i_ inhibited mineral deposition and the osteoblast‐related gene expression of human periodontal ligament stem cells (hPDLSC) and mouse pre‐osteoblast cells (MC3T3‐E1) via MAPK signaling as well as GPCR signaling pathways (Addison et al. 2007; Liang et al. 2021; Bourne et al. 2023).

In the local extracellular milieu, PP_i_ plays an inhibitory role in regulating biomineralization. However, the spontaneous inclusion of calcium‐PP_i_, considered an impurity phase in calcium phosphate (CaP) biomaterials, accelerates bone healing in the tibia defect model (Grover et al. 2013a). Currently, CaP biomaterials have been developed to explore alternative biomaterials in clinical dentistry, providing suitable conditions for dental pulp stem cells to promote dentin‐pulp repair and regeneration (Davaie et al. 2021). The small amount of calcium PP_i_ in biomaterials delays the dissolution of the material by inhibiting osteoclast activity. In this context, PP_i_‐incorporated biomaterial could also trigger osteoconductive potential (Le Gars Santoni et al. 2022). This discrepancy suggests PP_i_ can affect mineralization through multiple mechanisms.

SHED cell, referred to stem cell from human exfoliated deciduous teeth, is firstly separated from the pulp within a remnant crown of deciduous teeth (Miura et al. 2003). Specifically, SHED cells possess high proliferative, clonogenic, and immunomodulatory properties, as well as greater multipotency, compared with other MSCs, such as dental pulp stem cells (DPSC) and bone marrow mesenchymal stem cells (BM‐MSC) (Hosseini et al. 2025; Cordeiro et al. 2008; Sakai et al. 2010; Sabbagh et al. 2020; Kerkis et al. 2007; Wang et al. 2018; Winning et al. 2019). SHED highly maintains an immature characteristic with a positive expression of specific surface markers, such as neural crest and pluripotent markers (Miura et al. 2003; Kerkis et al. 2007). SHED have gained attention as a promising cell source for pulp‐dentin regeneration due to their multipotency, which allows them to differentiate into odontoblast‐like cells (Sabbagh et al. 2020; Farges et al. 2015). In addition, transplanted SHED cells and their secretome promote bone healing, rescue neurological disease, and trigger cartilage regeneration in rheumatoid arthritis (Miura et al. 2003; Mahdavi‐Jouibari et al. 2023; Huang et al. 2022). This evidence emphasizes the promising properties of SHED as a cell source in regenerative dentistry.

A challenge in understanding the functions of PP_i_ on cell differentiation is separating the direct effects of PP_i_ on cells from the indirect effects derived from the hydrolysis of PP_i_ and the production of P_i_ ions. Increased extracellular levels of P_i_ promote the expression of osteogenic marker genes and mineralization in SHED cells (Suwittayarak et al. 2024). However, the effects of PP_i_ on SHED cell function have not been determined. The present study aims to investigate the functions of PP_i_ in regulating SHED cells, particularly in the multipotential differentiation capacity. Transcriptomic profiling and pathway mechanisms were interrogated by RNA sequencing analysis.

## 2 Materials and Methods

### 2.1 Cell Culture

The cell isolation protocol was approved by the Human Research Ethical Committee, Faculty of Dentistry, Chulalongkorn University (No. 131/2023). Primary teeth collected for cell isolation were scheduled for extraction at the Department of Paediatric Dentistry according to the normal treatment plan. A cell explantation protocol was employed. Briefly, the minced dental pulp tissues were placed in high glucose Dulbecco's Modified Eagle Medium (Gibco, United States) supplemented with 10% fetal bovine serum (FBS) (cat. No. SV30160.03, Thermo Scientific, United States), 1% l‐glutamine (2 mM Glutamax TM‐1) (Thermo Scientific, USA), 1% of Penicillin (100 U/mL), Streptomycin (100 mg/mL) (Thermo Scientific, USA), and incubated at 37°C in a humidified 5% CO_2_ atmosphere. Upon reaching confluence, cells were subcultured using 0.25% trypsin/EDTA (cat. No. 25200‐072, Thermo Scientific, USA). Cells from passages 3‐7 were used in the experiments. The experiments were performed in four biological replicates (n = 4). Stem cell surface marker expression was analyzed using flow cytometry (BD FACS^Calibur^ and CellQuest software, BD Bioscience, San Jose, CA, USA) according to a previous report (Dominici et al. 2006).

Human peripheral blood mononuclear cells (PBMCs) were collected from a buffy coat of centrifuged human blood from the National Blood Centre, Thai Red Cross Society. The cell isolation protocol was approved by the Human Research Ethical Committee of the National Blood Centre (No. NBC 20/2021). The blood buffy coat was diluted by DPBS (Product code 16326239, Gibco, USA) containing 2% FBS. Then, the blood buffy coat solution was separated by overlaying 1: 1 mixture in Ficoll‐Paque Plus (GE17‐1440‐02, Cytiva, GE Healthcare, USA) and centrifuged at 450*g*, 25°C for 30 min. The middle buffy coat was pipetted and centrifuged at 450 g, 25°C for 10 min, four times. PBMCs were resuspended with minimum essential medium (⍺MEM, M4655, Sigma‐Aldrich, USA) containing 10% FBS and 1% penicillin (100 U/mL), streptomycin (100 mg/mL) before the experiment.

### 2.2 Reagents

Sodium pyrophosphate tetrabasic (Na_4_P_2_O_7_, PP_i_; cat. No. P8010‐500G, Sigma‐Aldrich, USA) was prepared as a 1 mM stock solution in serum‐free medium (SFM). The PP_i_ solution was diluted to 500 μM in SFM for experiments. Cells were treated with the following reagents: 1 and 10 μM PP_i_. For inhibition experiments, cells were incubated with an inhibitor for 30 min before PP_i_ exposure. The inhibitors were 30 μM ERK inhibitor (cat. No. 328006, Calbiochem, San Diego, CA, USA), 30 μM JNK inhibitor (SP600125, cas. No. 129‐56‐6, Sigma‐Aldrich, USA), 30 μM P38 inhibitor (SB239063, cat. No. 193551‐21‐2, Sigma‐Aldrich, USA), and 2.5 μM Pi3k inhibitor (Sigma‐Aldrich, USA).

### 2.3 Cytotoxicity Assay

Cells were incubated with 0.5 mg/mL MTT (Tocris Bioscience, UK) solution to demonstrate mitochondrial metabolism for 30 min in the incubator at 37°C in a humidified 5% CO_2_ atmosphere. The MTT solution was completely discarded, and the rest of the insoluble formazan was eluted with absolute DMSO, followed by measurement of the absorbance at 460 nm using a microplate reader (Biotek ELX800, USA), according to the manufacturer's protocol. These data were calculated as the percentage of viable cells.

### 2.4 Apoptosis Assay

Cells were detached using a 0.25% trypsin/EDTA solution, then centrifuged at 5000 rpm for 5 min. The supernatant was discarded, and the cell pellets were resuspended in 100 μL of 1× PBS. The 50 ng/mL propidium iodide (PI) (Cat. No. P4864, Sigma‐Aldrich, USA) was added, followed by 5 μL FITC annexin V reagent (Cat. No. 640945, Biolegend, USA). The mixture was kept in the dark for 15 min. Each tube was added with 400 μL of annexin V buffer (Cat. No. 640945, Biolegend, USA). Stained cells were analyzed with flow cytometry, according to the manufacturer's protocol.

### 2.5 Cell Cycle Analysis

Cells were detached using a 0.25% trypsin/EDTA solution, then centrifuged at 5000 rpm for 5 min. The supernatant was discarded, and the cell pellets were resuspended in 100 μL of 1× PBS and 350 μL of absolute ethanol. Cells were incubated at −20°C for 15 min. Then, the cells were centrifuged and washed twice with 2% PBS to collect the pellets. The pellet was resuspended in 500 μL of 1× PBS containing 4 mg/mL RNase solution (Sigma‐Aldrich, USA) and then incubated for 30 min. Finally, the cells were stained with 50 ng/mL propidium iodide (PI) and analyzed using flow cytometry, according to the manufacturer's protocol.

### 2.6 Colony‐Forming Unit Assay

Cells were seeded at a density of 5000 cells per well in a six‐well plate and cultured for 14 days. Cells were fixed with 10% formaldehyde (cat. no. F‐1268, Sigma‐Aldrich, USA) for 10 min and washed twice with PBS. Then, cells were stained with 0.6% crystal violet for 30 min and washed with PBS until the background was clear. Stained colony numbers were taken with a photo under the inverted microscope (Nikon ECLIPSE Ts2, Nikon, Melville, NY, USA), according to the manufacturer's protocol.

### 2.7 Cell Migration Assay

Cells were seeded at a density of 150,000 cells/well in a 12‐well plate overnight. The attached cell layer was disrupted with a sterile pipette tip before PP_i_ treatment to create a clear zone for measuring cell migration. Cells were monitored by taking photos using an inverted microscope at 0, 24, and 48 h. The width of the clear zone was measured using ImageJ software.

### 2.8 Mineralization Assay

Cells were seeded at a density of 25,000 cells in a 48‐well plate and cultured in osteogenic medium: Growth medium containing 50 µg/mL l‐ascorbic acid (cat. No. A‐4034, Sigma‐Aldrich, USA), 100 nM dexamethasone (cat. No. D8893, Sigma‐Aldrich, USA), and 5 mM β‐glycerophosphate (cat. No. G9422, Sigma‐Aldrich, USA). An osteogenic medium with varying PP_i_ concentrations was continuously treated and replaced with fresh medium every 2 days during the experiment. Osteogenic induction medium without PP_i_ was used as the control.

At Day 14, cells were fixed with 4% cold formaldehyde in PBS for 15 min. Then, the cells were washed twice with deionised water and stained with a 1% Alizarin Red S solution (cat. No. A5533, Sigma‐Aldrich, USA) for 5 min. Stained cells were washed with deionized water and dried at room temperature. Stained calcium nodules were observed under the inverted microscope. For quantification, the bound dye was eluted with 300 μL of a 10% cetylpyridinium chloride monohydrate solution and then measured at an absorbance of 570 nm using a microplate reader.

### 2.9 Intracellular Lipid Accumulation Assay

Cells were seeded at 25,000 cells/well in 48‐well plate and cultured for 21 days under adipogenic induction medium: Growth medium containing 500 μM 3‐Isobuty‐1‐methylxanthine (IBMX; cat. No. PH21124, Thermo Scientific, USA), 1 μg/mL insulin from bovine pancreas (Sigma‐Aldrich, USA), and 100 μM indomethacin (cat. No. I7378, Sigma‐Aldrich, USA). The medium was changed every 4 days. PP_i_ at different concentrations was maintained in the adipogenic medium throughout the experiment.

To measure intracellular lipid accumulation, cells were fixed with 4% cold formaldehyde in PBS for 15 min. Then, cells were gently washed twice with DI water. Thereafter, cells were incubated with 0.14% Oil Red O working solution for 10 min at room temperature. The solution was removed, and cells were gently washed with DI water. Stained cells were photographed under an inverted microscope.

### 2.10 Enzyme‐Linked Immunosorbent Assay (ELISA)

Conditioned medium was collected and stored at −20°C. The receptor activator of nuclear factor‐κB ligand (RANKL) concentration was determined using an ELISA kit (cat No. KGE004B, R&D Systems, USA), following the manufacturer's protocol.

### 2.11 Gene Expression Analysis

Cells were treated with 1 or 10 μM PP_i_ in growth medium for 1 day or in osteogenic medium for 7 days before collecting RNA. Gene expression was examined using quantitative real‐time polymerase chain reaction. Total cellular RNA was extracted using RiboExTM solution (cat. No. 301‐902, GeneAll, USA). The quality of RNA concentration was measured using Nanodrop (Thermo Scientific, USA). RNA was then converted into complementary DNA using an ImProm‐IITM Reverse Transcription System (cat. No. A3800, Promega, USA). One microliter of complementary DNA was used for real‐time polymerase chain reaction using a FastStart Essential DNA Green Master kit. The reaction was performed on a Bio‐Rad PCR system. Relative gene expression was calculated using the $2^{‐\Delta \Delta C_{t}}$ method. *GAPDH* was used as a reference gene. The expression value was normalized to the *GAPDH* expression value and the control. The oligonucleotide primers used for this study are in Table S1.

### 2.12 Osteoclast Differentiation

PBMCs were seeded at 1 × 10^6^ cells/well in 48‐well plates to allow the cells to attach overnight. Attached PBMC cells were incubated with α‐MEM medium containing 10% FBS, 50 ng/mL soluble RANKL (Biolegend, USA), and 30 ng/mL MCS‐F (Biolegend, USA) for 10 days. The 12% conditioned medium derived from the control or PP_i_‐treated SHED was co‐cultured with an attached PBMC cell culture. Attached PBMC cells cultured without the conditioned medium treatment were used as the control.

Cells were fixed with 4% formaldehyde in PBS for 15 min. Then, cells were gently washed twice with PBS. TRAP Basic incubation medium was prepared with 320 g of sodium acetate anhydrous (Sigma‐Aldrich), 1.14 g of L‐(+) tartaric acid (Sigma‐Aldrich, USA), 280 g of glacial acetic acid, and 100 mL of DI water, adjusting the pH to 4.7–5.0 with NaOH or glacial acetic acid. Multi‐nucleated cells were incubated for 60 min in an incubator with freshly prepared TRAP staining solution containing 1 mL TRAP Basic incubation medium, 5 μL naphthol AS‐MX phosphate substrate (20 mg naphthol AS‐MX phosphate (Sigma‐Aldrich, USA), 1 mL ethylene glycerol monoethyl ether (Sigma‐Aldrich, USA)), and 0.6 mg fast red violet LB salt. Thereafter, the cells were washed with deionised water and counterstained with 0.02% fast green (CAS 2353‐45‐9, Sigma‐Aldrich, USA) in deionised water for 30 min. The cells were preserved in a 50% glycerol solution to be photographed under inverted microscopy. A TRAP‐positive multinucleated (3 or more nuclei) cell was defined as an osteoclast‐like cell (Ghayor et al. 2011) and counted under inverted microscopy.

### 2.13 Gene Expression Profile Analysis

Global differential gene expression was performed at the Omics Sciences and Bioinformatics Centre, Faculty of Science, Chulalongkorn University. Cells were treated with 10 μM PP_i_ for 24 h in a growth medium. Total RNA was isolated using a RNeasy kit (Cat. No. 74104, Qiagen, USA). DNase treatment was performed in columns. The total RNA quantity and quality were examined using a Nanodrop and an Agilent 2100 Bioanalyzer system. Library quality assurance was conducted using an Agilent 2100 Bioanalyzer system and Qubit 3.0 fluorometer. Sequencing was performed in a NextSeq. 500 sequencing system. Read quality was checked, trimmed, and filtered with a FastQC and FastX toolkit (Andrews 2018). Reads were mapped with *Homo sapiens* UCSC hg38 using TopHat2 (Kim et al. 2013; Trapnell et al. 2012). FPKM estimation of reference genes and transcripts was performed using DeSeq. 2 analysis. Differentially expressed genes were analyzed for function and pathway using network‐based visual analytics for gene expression profiling, meta‐analysis, and interpretation, NetworkAnalyst (Xia et al. 2015). Sequencing data were submitted to the NCBI's Gene Expression Omnibus (GSE281644).

### 2.14 Statistical Analyses

Data are shown on graphs as mean ± SD. Statistical significance was evaluated using a non‐parametric model, the Mann–Whitney *U* test. *p*‐values < 0.05 were considered significant. All statistical analyses were performed using Prism 9.01 software (GraphPad software).

### 2.15 Declaration of AI‐Assisted Technologies in the Writing Process

During the preparation of this study, the authors utilized AI‐assisted technologies to improve readability and language. After using this tool/service, the authors reviewed and edited the content as needed and take full responsibility for the content of the publication.

## 3 Results

### 3.1 PP_i_ Regulates SHED Cell Proliferation, Apoptosis, and Migration

**Figure 1:**
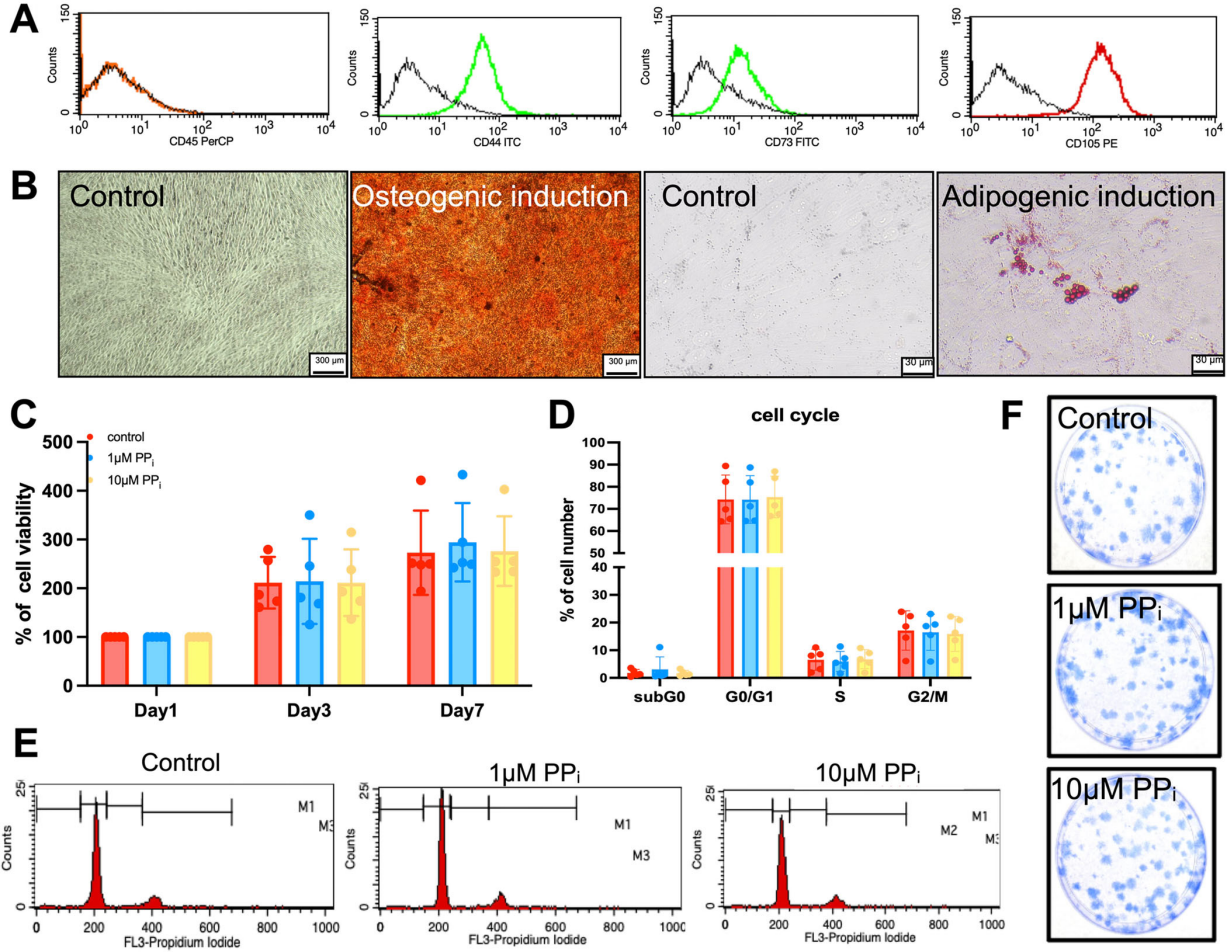
PP_i_ regulates SHED cell proliferation. Stem cell surface markers were examined using flow cytometry analyses (A). Mineral deposition and intracellular lipid accumulation were determined using Alizarin Red S staining and Oil Red O staining, respectively (B). Cell viability was determined by MTT assay (C). Cell cycle progression analysis at Day 3 was determined using flow cytometry analysis (D, E). Colony‐forming unit capability was evaluated by Coomassie blue staining on Day 14 (F).

SHED were characterized for MSC phenotypes based on minimal criteria for multipotent stem cells (Dominici et al. 2006). The isolated cells expressed MSC surface markers (CD44, CD73 and CD105) while lacking a hematopoietic surface marker (CD45) (Figure 1A). When cultured in appropriate conditions, cells were able to differentiate into osteogenic and adipogenic lineages, as confirmed by mineral deposition and intracellular lipid accumulation, respectively (Figure 1B).

The percentage of cell viability, assessed by MTT assay, showed no differences between control, 1 μM, or 10 μM PP_i_ at Days 1, 3, and 7 (Figure 1C). To determine the influence of PP_i_ on cell cycle progression of SHED cells, the DNA content, stained by propidium iodide (PI), was assessed by flow cytometry (Figure 1D,E). There were no significant differences in the percentage of cells in the G0/G1, S, and G2/M phases comparing between the control, 1 μM, and 10 μM PP_i_ groups, demonstrating that PP_i_ did not alter cell proliferation. Regarding SHED cell colony‐forming ability, 1 μM or 10 μM PP_i_ did not alter the number of colonies, compared to the control (Figure 1F).

**Figure 2:**
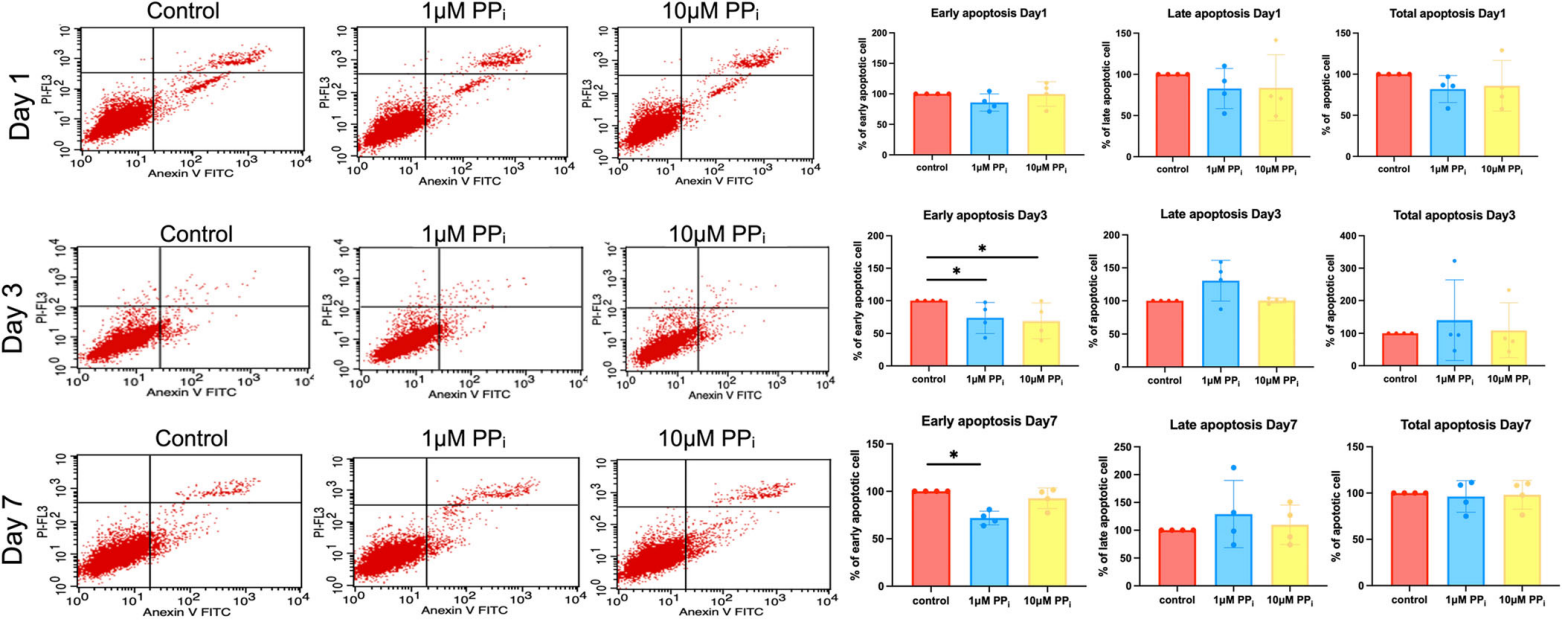
PP_i_ regulates SHED cell apoptosis. Cell apoptosis was determined using Annexin V FITC/PI staining at Days 1, 3, and 7. The scattered plots and the percentage of apoptotic cells are illustrated. Bars indicate a statistically significant difference. *p < 0.05 compared to the control.

Apoptosis was determined by staining with annexin V and propidium iodide (PI). By Day 1, PP_i_ did not alter the proportion of annexin V^+^/PI^−^ (early) and annexin V^+^/PI^+^ (late) apoptotic cells (Figure 2). However, on Day 3, both 1 and 10 μM PP_i_ reduced the proportion of the early apoptotic cells to approximately 80% and 75%, compared to the control. On Day 7, 1 μM PP_i_ significantly reduced the proportion of the early apoptotic population of SHED cells to 80% (*p* < 0.05), suggesting that PP_i_ reduced early apoptosis (Figure 2). Late and total proportions of apoptotic cells did not differ between the control and PP_i_‐treated groups at any time point (Figure 2).

**Figure 3:**
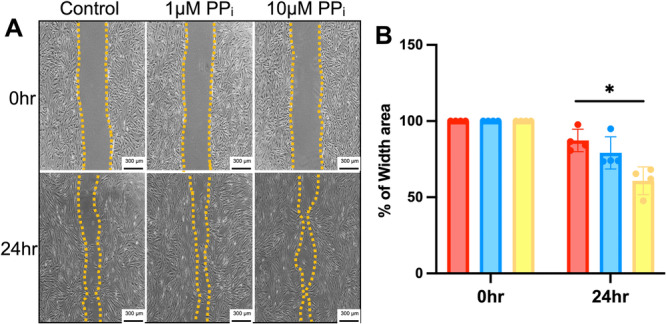
PP_i_ regulates SHED cell migration. A scratch assay was employed to evaluate cell migration (A), and the percentage of the space area was determined using ImageJ (B). Bars indicate a statistically significant difference. *p < 0.05 compared to the control.

The scratch assay was used to investigate the potential effects of PP_i_ on cell migration (Rodriguez et al. 2005). At 24 h, the gap space was slightly decreased in cells treated with 1 μM PP_i_, but the 10 μM PP_i_‐treated group showed a significant reduction of space compared to the control (Figure 3A,B) (*p* < 0.05), indicating that higher PP_i_ induced an *in vitro* SHED cell migration.

### 3.2 PP_i_ Attenuates Mineralization and Regulates Gene Expression in SHED Cells

**Figure 4:**
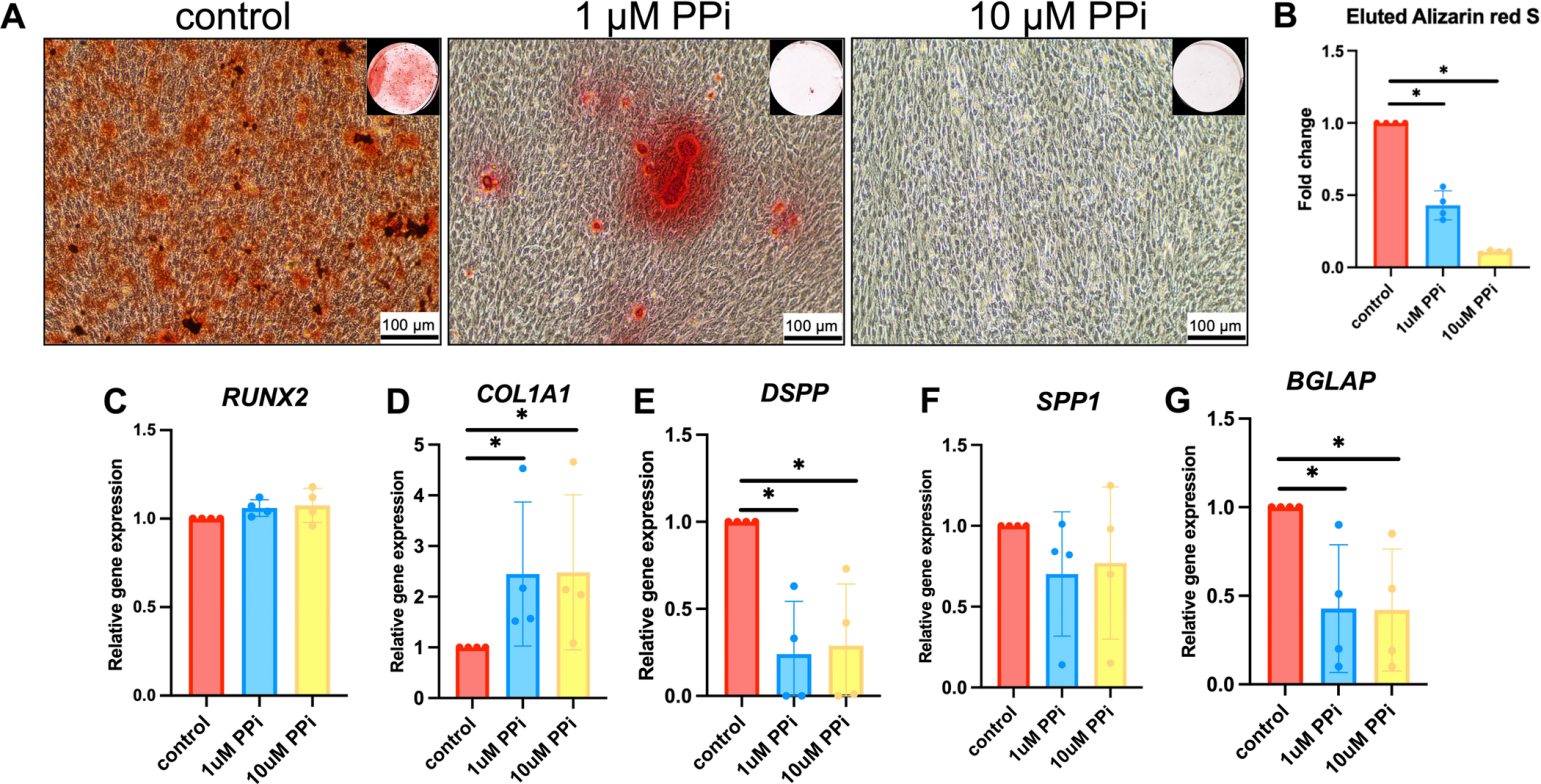
PP_i_ attenuates mineralization and regulates gene expression in SHED cells. SHED cells were treated with PP_i_ in osteogenic induction medium. Mineral deposition was assessed using Alizarin Red S staining on Day 14 (A). Quantitative analysis of eluted Alizarin Red S dye was measured at an absorbance of 570 nm (B). Gene expression was determined using real‐time polymerase chain reaction at Day 7 (C–G). Bars indicate a statistically significant difference. *p < 0.05 compared to the control.

To test the effects of PP_i_ on mineralization, SHED cells were cultured in an osteogenic induction medium for 14 days. Mineral deposition was decreased in a dose‐dependent manner in PP_i_‐treated cells (Figure 4A). Both 1 and 10 μM PP_i_ significantly inhibited mineralization in SHED cells by more than 50% and 80%, respectively (Figure 4B) (*p* < 0.05).

Odontoblast‐associated gene expression was tested by qPCR. At Day 7, SHED cells treated with PP_i_ upregulated *COL1A1* mRNA expression by more than 100% while simultaneously downregulating *DSPP* and *BGLAP* by more than 75% and 50%, respectively (Figure 4C–G) (*p* < 0.05). Molecular pathways underlying gene expression changes were interrogated by pretreating SHED cells with ERK, p38, JNK, or PI3K inhibitors for 30 min before PP_i_ exposure, pathways implicated in odontoblast differentiation and/or mineralization (Addison et al. 2007; Liang et al. 2021; Doan et al. 2012). ERK, JNK, and PI3K inhibitors failed to rescue the effect of PP_i_‐attenuated mineralization in SHED cells (Figure S1). Pretreatment with p38 inhibitor caused a small but significant increase in mineral deposition compared to PP_i_‐treated cells; however, this did not substantially ameliorate the inhibitory effect of PP_i_ on mineralization (Figure S1D).

### 3.3 PP_i_ Decreases Adipogenic Differentiation Potency in SHED Cells

**Figure 5:**
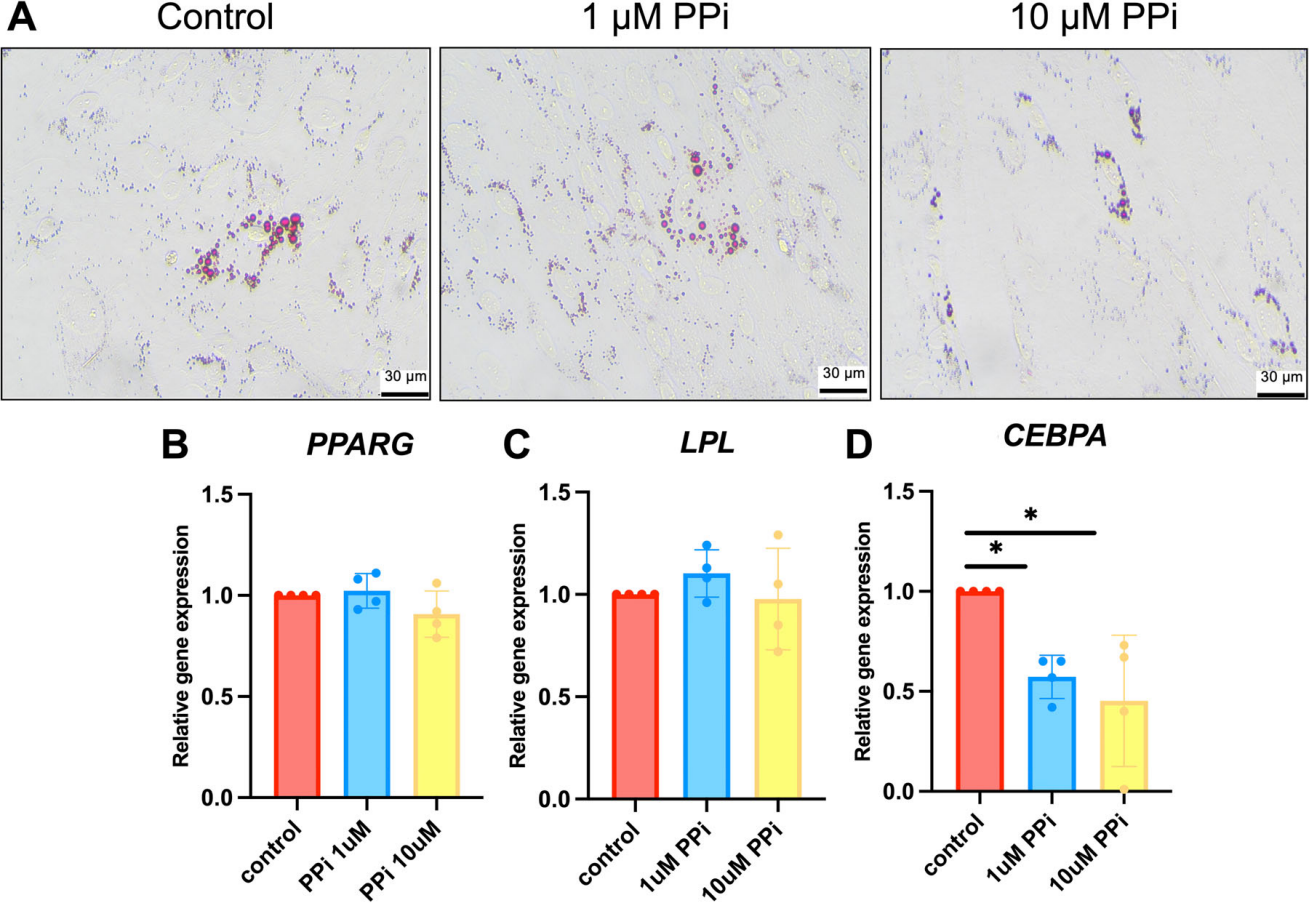
PP_i_ decreases adipogenic differentiation potency in SHED cells. SHED cells were treated with PP_i_ and maintained under adipogenic induction medium. At Day 16, intracellular lipid accumulation was assessed using Oil red O staining (A). Gene expression was determined using real‐time polymerase chain reaction at Day 7 (B–D). Bars indicate a statistically significant difference. *p < 0.05 compared to the control.

A previous study reported that the indirect increase in PP_i_ concentration induced by levamisole, a TNAP inhibitor that reduces P_i_ ion accumulation, led to the gene expression of adipogenic markers in periodontal ligament stem cells (PDLSCs) (Melms et al. 2020). In SHED cells cultured in adipogenic media, PP_i_ slightly suppressed intracellular lipid accumulation (Figure 5A). Analyzing expression of adipogenic markers at Day 8 by qPCR demonstrated that *PPARG* and *LPL* mRNA levels did not change in PP_i_‐treated groups (Figure 5B,C). However, *CEBPA* mRNA levels decreased by approximately 50% in the 1 and 10 μM PP_i_‐treated groups compared to the control (Figure 5D) (*p* < 0.05).

### 3.4 PP_i_ Indirectly Attenuates SHED Cell Induction of Osteoclast Formation

**Figure 6:**
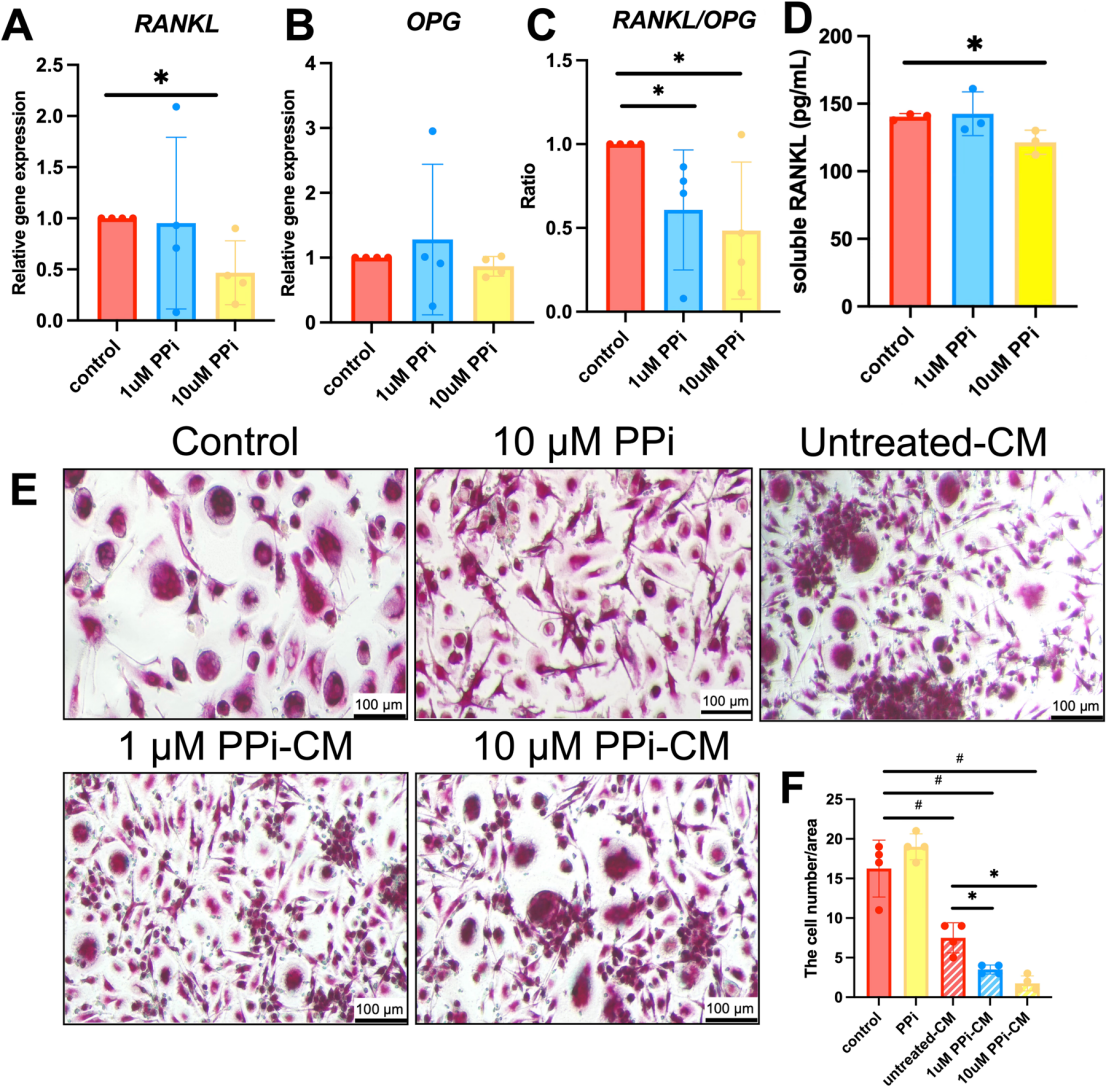
PP_i_ indirectly attenuates SHED cell induction of osteoclast formation. SHED cells were treated with PP_i_ for 24 h in a growth medium. Gene expression was determined using real‐time polymerase chain reaction (A–B). The *RANKL/OPG* expression ratio was calculated (C). Soluble RANKL was examined using ELISA (D). PBMCs were cultured in the presence of MSCF and RANKL. Then, cells were exposed to conditioned medium (CM) from SHED culture in the control untreated condition and PP_i_‐treated conditions (E). PBMCs cultured in fresh medium (control) and fresh medium supplemented with 10 μM were used as the control. The number of TRAP‐positive, multinucleated cells was counted (F). Bars indicate a statistically significant difference. *p < 0.05 compared to control or untreated conditioned medium derived from control. ^#^ *p* < 0.05 compared to osteoclast control.

Osteoblasts employ RANKL and osteoprotegerin (OPG) to regulate differentiation and activation of osteoclasts to resorb bone. Effects of PP_i_ on SHED expression of *RANKL* and *OPG* were examined after 24 h of PP_i_ treatment. *RANKL* mRNA levels decreased by 50% in the 10 μM PP_i_ treatment group compared to the controls (Figure 6A) (*p* < 0.05). However, there was no change in *OPG* mRNA levels between groups (Figure 6B). Hence, the *RANKL/OPG* ratio was significantly decreased as a result of PP_i_ treatment (Figure 6C) (*p* < 0.05). Measurement of soluble RANKL secreted into the conditioned medium showed that 10 μM PP_i_ decreased soluble RANKL levels, consistent with gene expression results (Figure 6D) (*p* < 0.05).

To determine the functional effects of PP_i_ treatment of SHED cells on osteoclast differentiation, the conditioned medium (CM) from control and PP_i_‐treated SHED cells was collected and transferred to PBMC culture. MCS‐F and RANKL were added to induce osteoclast differentiation. PBMCs treated with CM from PP_i_‐treated SHED cells exhibited lower osteoclast numbers compared to cells treated with CM from the control condition (Figure 6E,F) (*p* < 0.05).

### 3.5 PP_i_ Alters SHED Cell Transcriptome

**Figure 7:**
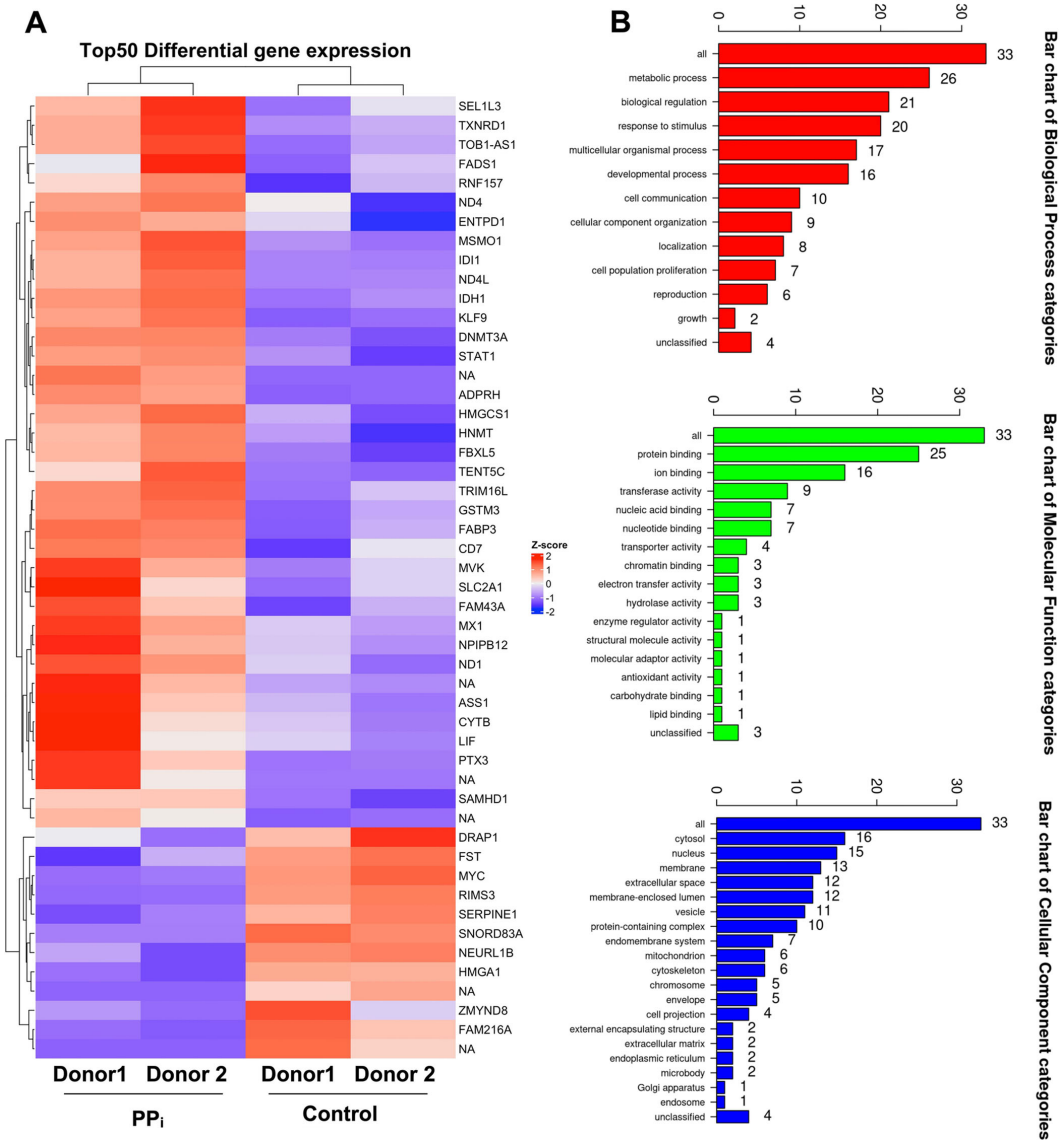
PP_i_ alters SHED cell transcriptome. SHED cells were treated with 10 μM PP_i_ for 24 h in growth medium. The transcriptomic profile was examined using RNA sequencing analysis. The top 50 differentially expressed genes were illustrated using a heatmap (A). Gene ontology analysis was demonstrated (B).

Our study revealed that PP_i_ modulates the behaviors of SHED cells, including cell migration, cell death, and their multipotential differentiation ability. To interrogate gene expression changes on a large and more agnostic scale, we performed transcriptomic profiling of SHED treated with PP_i_ under growth medium for 1 day. The top 50 significantly expressed genes are shown in a heat map, demonstrating larger and consistent differences in global gene expression between PP_i_‐treated and control SHED cells (Figure 7A).

**Figure 8:**
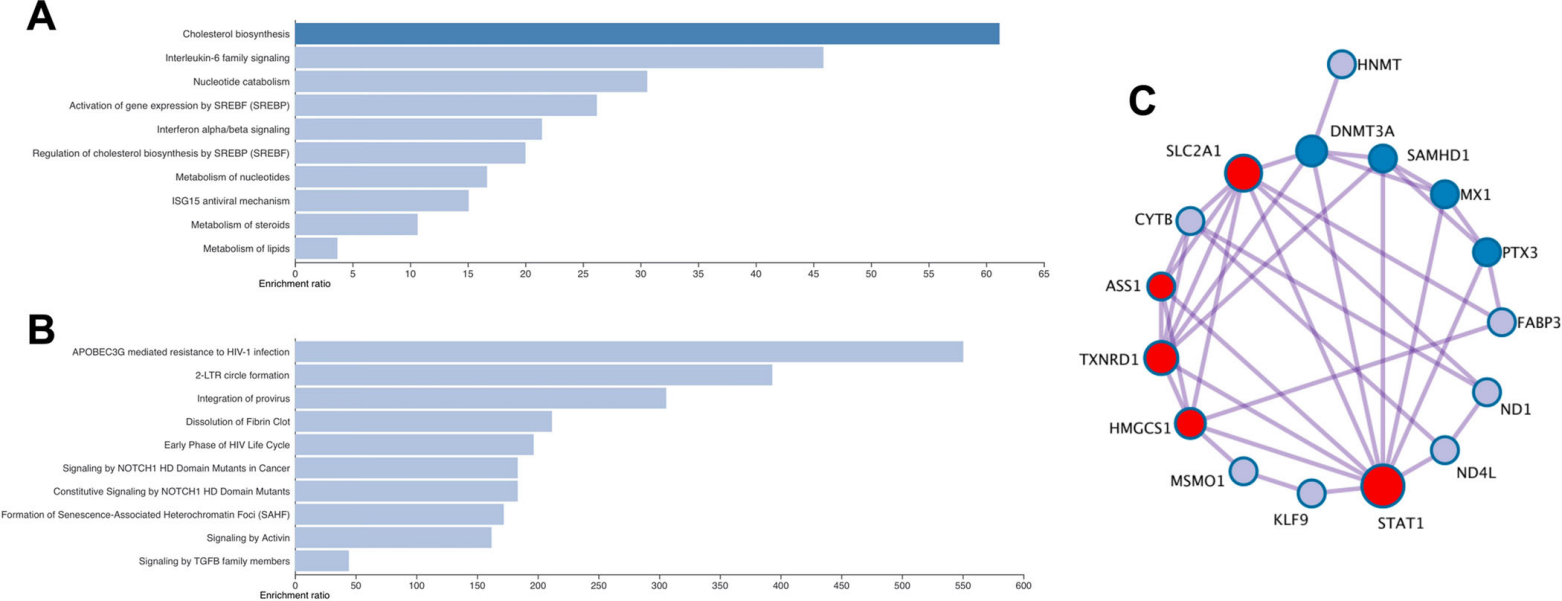
PP_i_ alters SHED cell transcriptome. KEGG pathway enrichment for upregulated (A) and downregulated (B) genes is demonstrated. Metascape analysis for protein‐protein interaction is shown (C).

Gene ontology (GO) analysis was employed to analyze GO categories, including biological function, cellular component, and molecular function. Metabolic processes and protein binding groups contained the highest numbers of altered genes, as shown in Figure 7B. Significantly enriched pathways were identified using KEGG database analysis. PP_i_ upregulated genes involved in pathways of cholesterol biosynthesis, interleukin‐6 family signaling, and interferon alpha/beta signaling (Figure 8A). Significantly downregulated genes included those involved in signaling by NOTCH1 HD domain mutants in cancer, constitutive NOTCH1 HD domain mutants, and signaling by TGF‐beta family members (Figure 8B). Metascape analysis displayed the protein‐protein interaction (PPI), demonstrating the association with interferon alpha/beta signaling, interferon signaling, and metabolism of lipids, as shown in Figure 8C.

## 4 Discussion

PP_i_ plays a crucial regulatory role in both physiological and pathological mineralization. PP_i_ acts as a potent inhibitor of tissue mineralization by blocking hydroxyapatite (HA) crystal nucleation and growth (Fleisch et al. 1966). In human plasma, PP_i_ is optimally maintained at a concentration range of 1–10 μM to prevent unwanted mineralization in vascular tissues and joints (Silcox and McCarty 1973; Russell et al. 1971b; Rachow et al. 1988). Conversely, P_i_ circulates at mM concentrations, making PP_i_ levels about 0.5% of P_i_ levels, yet still able to maintain normal calcification patterns in tissues (Bisaz et al. 1968). Excessive PP_i_ levels, exceeding 10 μM, increase the risk of both inhibiting normal skeletal and dental mineralization, as well as inducing the precipitation of calcium dihydrate pyrophosphate (CPP) crystals, leading to pathological calcification (Zhou et al. 2012; Rachow et al. 1988).

This has relevance in contexts of normal mineralized tissue development and maintenance when ANKH, ENPP1, or TNAP expression changes in local cells, as well as in pathological situations where loss‐of‐function in one of these factors alters levels of PP_i_ outside the physiologic range. Intracellular PP_i_ or ATP, a PP_i_ precursor, is exported outside the cell via actions of ANKH, a transmembrane protein (Ho et al. 2000; Szeri et al. 2020). PP_i_ can be rapidly generated from ATP by the ectoenzyme, ENPP1 (Harmey et al. 2004). Increasing extracellular PP_i_ concentrations acts as a potent inhibitor for tissue calcification (Orriss et al. 2016; Russell et al. 1971a). To facilitate skeletal and dental tissue mineralization, PP_i_ levels are locally reduced by the expression of TNAP by mineralizing cells, which produces P_i_ ions through the hydrolysis of ATP, UTP, phosphate‐binding proteins, and PP_i_ (Thouverey et al. 2009). TNAP loss‐of‐function, as seen in the inherited metabolic error of hypophosphatasia (HPP), causes the accumulation of excess extracellular PP_i_ concentrations, impairing bone and tooth mineralization (Millán and Whyte 2016; Foster et al. 2024). In addition, cytosolic inorganic pyrophosphatase (PP_i_‐ase) also generates intracellular P_i_ to maintain its concentration and preserve cellular metabolism (Shaw et al. 1989; Polewski et al. 2010). The influence of intracellular PP_i_ concentrations on mineralization remains unclear (Harmey et al. 2004).

In addition to the well‐established function of PP_i_ in controlling tissue mineralization by inhibiting HA nucleation and growth, there is accumulating evidence for a broader scope of effects of PP_i_ on cell functions. It is essential to understand the other functions of PP_i_ in regulating cellular activities for these reasons. However, systemic PP_i_ concentrations are typically maintained within the low micromolar range; the precise local concentration within tissues is difficult to measure due to the dynamic regulation (Bisaz et al. 1968). In the present study, we selected PP_i_ concentrations of 1 and 10 µM for our experiments, based on a previous study that has determined the role of PP_i_ in regulating cell responses (Nowwarote et al. 2018). These doses fall within the range reported in circulation and thus provide a reasonable approximation for studying the cellular responses of SHED under conditions that may reflect physiological exposure. We found that PP_i_ at 1 or 10 μM regulated SHED cell apoptosis, migration, mineralization, cell fate, and transcriptome. These profound effects strongly support that molecular mechanisms other than HA inhibition are at play when cells are exposed to PP_i_.

In our study, PP_i_ was likely to enhance cell migration and inhibit cell apoptosis in SHED cells. Recently, cell migratory ability has been associated with the interaction of cell‐to‐cell or cell‐to‐matrix adhesion molecules such as N‐cadherin (Shih and Yamada 2012). N‐cadherin‐deficient cells contribute to the inhibition of cell migratory ability (Klingener et al. 2014). Additionally, the stabilization of N‐cadherin and fibroblast growth factor receptor (FGFR) has been reported to inhibit cell migration (Nguyen et al. 2019). In addition, an increase in N‐cadherin expression prevents cell apoptosis (Osuka et al. 2021). One possible explanation is that PP_i_ may promote cell migratory ability and inhibit cell apoptosis by regulating N‐cadherin, while simultaneously inactivating N‐cadherin and FGFR through a decrease in intracellular P_i_, which is required for phosphorylation (Osuka et al. 2021; Ito et al. 2007; Nishino et al. 2017). While speculative, these SHED cell responses documented here may be part of a PP_i_‐driven mechanism that is part of a stem cell mobilization in response to trauma or injury, *in vivo*.

We did confirm the strong inhibition of mineralization by SHED cells by physiological doses of 1 and 10 μM doses of PP_i_ (Bisaz et al. 1968). Our previous study has shown that 10 μM PP_i_ inhibited *in vitro* mineralization by SHED cells (Nowwarote et al. 2018). Other studies have documented the inhibitory action of PP_i_ on mineralization by osteoblasts, cementoblasts, and periodontal ligament cells (Foster et al. 2012; Addison et al. 2007; Liang et al. 2021). However, PP_i_ regulates mineralization through multiple mechanisms. PP_i_ tightly binds to the surface of hydroxyapatite crystals, blocking crystal growth (Addison et al. 2007; Fleisch et al. 1966). PP_i_ can also induce expression of osteopontin (OPN), an extracellular matrix protein, which also acts as a mineralization inhibitor (Addison et al. 2007; Yuan et al. 2014). We did not find a significant effect on *SPP1*/OPN expression when SHED cells were exposed to 1 or 10 μM PP_i (_Foster et al. 2018_)_. However, both 1 and 10 μM PP_i_ increased type I collagen (*COL1A1*) expression and simultaneously decreased expression of dentin sialophosphoprotein (*DSPP*) and osteocalcin (*BGLAP*) (Bourne et al. 2023; Polewski et al. 2010; Nowwarote et al. 2018). The *DSPP* gene gives rise to dentin sialoprotein (DSP) and dentin phosphoprotein (DPP), ECM proteins closely related to OPN, which promote dentin mineralization (Liang et al. 2023). BGLAP is also a well‐established dentin extracellular matrix protein, and when carboxylated, it promotes mineralization (Papagerakis et al. 2002). Other studies have reported potential signaling effects of PP_i_ on mineralizing cells (Foster et al. 2012; Pujari‐Palmer et al. 2016; Addison et al. 2007; Liang et al. 2021; Bourne et al. 2023; Grover et al. 2013b; Abdelmagid et al. 2020). Consistent with established evidence, the inhibition of mineralization in response to PP_i_ was ameliorated by adding a P38 inhibitor (Pujari‐Palmer et al. 2016; Addison et al. 2007). Although previous studies have implicated MAPK signaling pathways in the inhibition of PPi on osteogenic‐related gene expression, no study has demonstrated a role for these pathways in the mineralization process.

Currently, regulatory P_i_/PP_i_ effectors also modulate the cell fate of adipogenesis. For example, BMMSCs, bone marrow mesenchymal stem cells, favored adipogenic differentiation and lipid formation in the *ank*‐knockdown model (Minashima et al. 2017). TNAP inhibition promoted *LPL* gene expression in PDLSCs and DPSCs (Melms et al. 2020). We initially established the direct effects of PP_i_ on the adipogenic capacity of SHED, demonstrating that the presence of PP_i_ resulted in decreased *CBEP/α* mRNA expression, consistent with a previous report (Minashima et al. 2017). The earlier expression of LPL and CBEP/⍺ is required to drive cell fate to adipogenesis (Rosen 2005). This finding suggests that PP_i_ plays a key role in the determination of adipogenic fate.

We further documented the indirect effect of PP_i_ on osteoclast activation for the first time in SHED cells. The higher dose of 10 μM PP_i_ modulated the differentiation and activity of bone‐resorbing osteoclasts by reducing RANKL expression and secretion. This translated to significantly fewer TRAP+ osteoclasts *in vitro* when conditioned media from PP_i_‐treated SHED cells were added to PBMCs during osteoclastic differentiation. However, there have been conflicting reports regarding the effects of PP_i_ on osteoclast activity. PP_i_ concentrations greater than 1 mM induced TRAP mRNA and protein expression. Additionally, a higher PP_i_ concentration increased the number of TRAP‐positive osteoclast‐like cells (Abdelmagid et al. 2020). Moreover, co‐culture between osteoblasts and osteoclasts during PP_i_ treatment has been shown to enhance osteoclast differentiation and activity in a prior study (Abdelmagid et al. 2020). A dose of 100 μM PP_i_ decreased the activity and size of osteoclast cells, but these effects were not found for 10 μM PP_i_, consistent with our finding demonstrating that direct treatment of 10 μM PP_i_ to PBMC did not affect the number of TRAP‐positive multinucleated cells (Bourne et al. 2023). Conditioned medium derived from untreated SHED cells reduced the TRAP‐positive multinucleated cell formation by PBMC culture. In this regard, SHED expresses several molecules regulating osteoclast differentiation, including RANKL and OPG.

We expanded our analysis beyond qPCR to identify additional transcriptomic changes in SHED cells resulting from PP_i_ treatment. PP_i_ differentially upregulated genes in cholesterol and lipid metabolism and inflammation signaling pathways. Cholesterol is associated with bone metabolism. A previous study demonstrated that lipid metabolism reduced ROS activity in ovariectomized mice (Li et al. 2024). An increase in lipid and cholesterol metabolism supported the differentiation toward adipogenesis but not osteogenesis (Zhang et al. 2024). The present study observed an inhibitory effect of PP_i_ on *CBEP/⍺* expression. Intracellular lipid accumulation was slightly decreased, but there was no marked difference. The role of PP_i_ in adipogenesis requires further investigation *in vivo*. As for inflammation, STAT1 is a signal transducer for interferon signaling. Our studies indicated that STAT1 was upregulated in PP_i_‐treated conditions. STAT1 is associated with a decrease in bone volume and impaired osteogenic differentiation (Sunhwa et al. 2003; Zhang et al. 2022). However, an increase in lipid metabolism and inflammation has been reported to be associated with osteoclast formation and its activity. Based on our transcriptomic analysis, the expression of *LDL* was significantly upregulated in response to PP_i_. High LDL combined with pro‐inflammatory cytokine abolishes the formation of osteoclasts (Ascone et al. 2020). Consistently, the indirect effect of PP_i_ strongly decreased the number of osteoclasts. Functional experiments are still required to clarify the relevant molecule regulating osteoclastogenesis.

Furthermore, we observed that the differentially downregulated genes were enriched in the NOTCH D1 and TGF‐β family signaling pathways. These pathways are a main commitment for osteogenic differentiation in MSC (Aval et al. 2017). NOTCH signaling requires cell‐to‐cell interaction to transmit the signal transduction (Kopan and Ilagan 2009). The study has established that the NOTCH signal could cross‐talk with the TGF/BMP‐9 signaling pathway in regulating early and later osteogenic stages (Cao et al. 2017). The TGF family is abundant in several tissues, playing a crucial role in modulating tissue homeostasis. Among TGF family members, TGF‐β is an essential inducer for osteoblast differentiation (Wei et al. 2024). These data suggest that PP_i_ may inhibit the osteogenic ability of SHED by increasing the interferon signaling pathway and decreasing the NOTCH and TGF signaling pathways. However, further in‐depth analysis of differentially expressed genes in these pathways is required to confirm this hypothesis.

Several limitations must be noted in the present study. As discussed above, the concentration of PP_i_ in both physiological and pathological conditions in humans remains a topic of controversy and ambiguity. Hence, the concentration studied may be directly related to the *in vivo* levels. The discrepancy in dosage may lead to different outcomes *in vivo*. Furthermore, we recruited four representative donors for the present study. Although the statistical analysis indicated a significant difference, it is worth noting that variations in biological background can influence cell responses. In addition, the biological pathways identified in the present study from the transcriptome profile have not yet been confirmed through a functional study. Further study should be conducted to elucidate the in‐depth cellular mechanism underlying the control of cell responses by PP_i_.

In conclusion, PP_i_ modulates cell differentiation of SHED. In this regard, PP_i_ inhibits mineralization and osteogenic differentiation. PP_i_ attenuates adipogenic differentiation by decreasing *CEBPA* mRNA expression. Further, PP_i_ decreases RANKL expression and subsequently indirectly modulates osteoclast differentiation. This study provides a deeper understanding of the regulatory mechanisms underlying the effects of PP_i_ on SHED cells.

## Author Contributions

Ravipha Suwittayarak contributed to experimental design, data acquisition, data interpretation, and manuscript drafting. Nunthawan Nowwarote and Chatvadee Kornsuthisopon were involved in data acquisition and interpretation. Waleerat Sukarawan and Brian L. Foster contributed to data interpretation. Hiroshi Egusa and Thanaphum Osathanon contributed to conceptual design, project management, and data interpretation. All authors critically revised the manuscript and approved it for publication.

## Ethics Statement

The present study was approved by the Human Research Ethics Committee of the Faculty of Dentistry, Chulalongkorn University, Bangkok, Thailand. Written informed consent was obtained from patients/participants before their participation in this study. The study adhered to the Declaration of Helsinki.

## Consent

The authors have nothing to report.

## Conflicts of Interest

The authors declare no conflicts of interest.

## Supporting information


**Supporting Figure 1:** SHED cells were cultured in an osteogenic medium for 14 days. In the inhibitor condition, cells were exposed to the inhibitor for 30 min prior to PP_i_ exposure. Mineral deposition was evaluated using Alizarin Red S staining, and the absorbance of eluted dye at 570 nm was demonstrated in the graph. Bars indicate a statistically significant difference. *p < 0.05, compared to control. ^#^ *p* < 0.05, compared to the PP_i_‐treated condition.

**Supporting Table 1:** Oligonucleotide sequence.

## Data Availability

The data that support the findings of this study are openly available in NCBI's Gene Expression Omnibus repository at https://www.ncbi.nlm.nih.gov/geo/query/acc.cgi?acc=GSE281644, reference number GSE281644.
