# Supplementary material for: Pyrophosphate Regulates Multilineage Differentiation in Stem Cells From Human Exfoliated Deciduous Teeth
Source: Clin Exp Dent Res. 2025 Nov 20;11(6):e70248. doi: 10.1002/cre2.70248 (PMC12631897; doi:10.1002/cre2.70248)
Supplement: Supplementary file 1 — Supporting Figure 1: SHED cells were cultured in an osteogenic medium for 14 days. In the inhibitor condition, cells were exposed to the inhibitor for 30 min prior to PPi exposure. Mineral deposition was evaluated using Alizarin Red S staining, and the absorbance of eluted dye at 570 nm was demonstrated in the graph. Bars indicate a statistically significant difference. *p < 0.05, compared to control. # p < 0.05, compared to the PPi‐treated condition. [file CRE2-11-e70248-s001.docx]

**
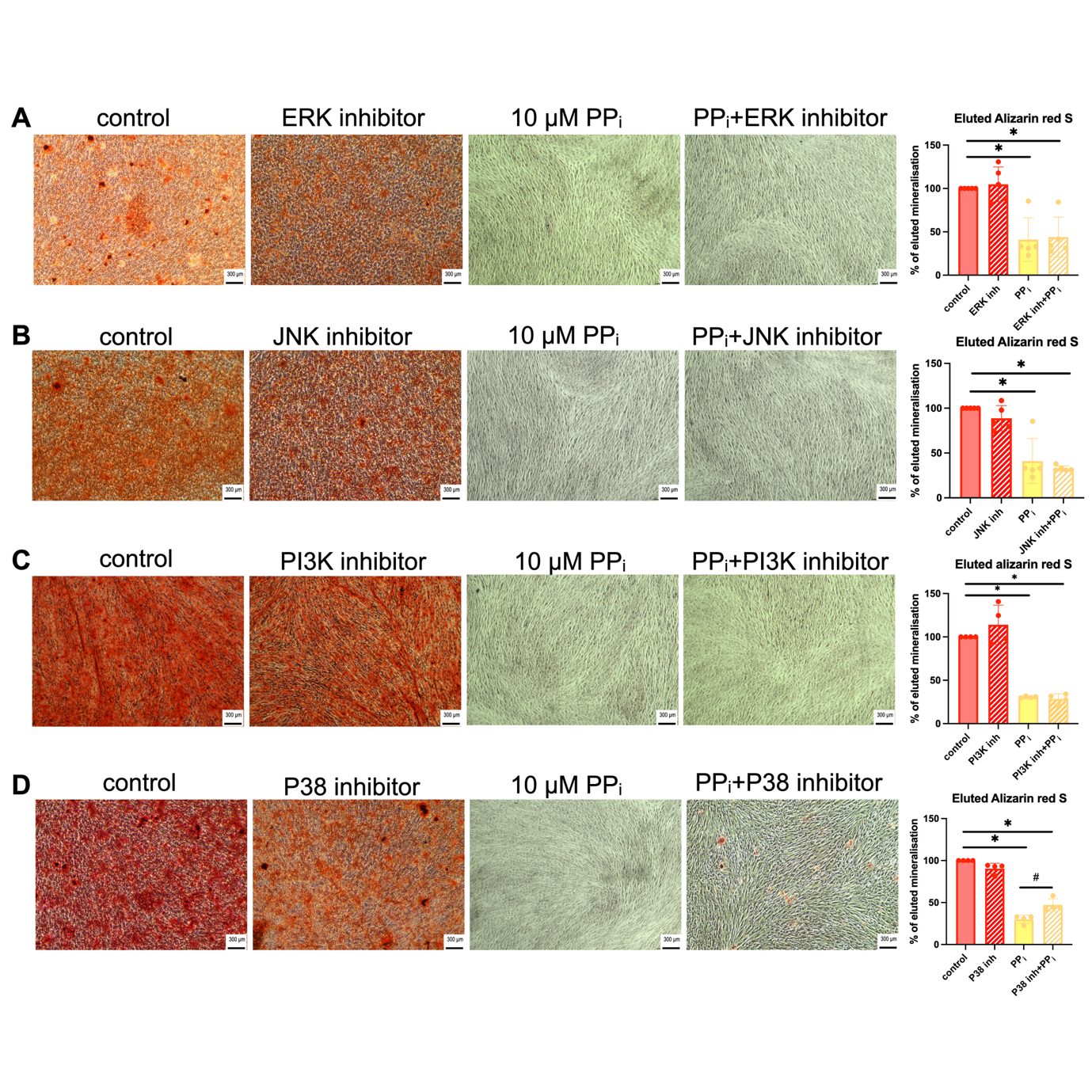
**

**Supplementary Figure 1.** SHED cells were cultured in an osteogenic medium for 14 days. In the inhibitor condition, cells were exposed to the inhibitor for 30 min prior to PP_i_ exposure. Mineral deposition was evaluated using Alizarin Red S staining, and the absorbance of eluted dye at 570 nm was demonstrated in the graph. Bars indicate a statistically significant difference. ^*^*P* < 0.05, compared to control. ^#^*P* < 0.05, compared to PP_i_ treated condition.
