# Supplementary material for: Pyrophosphate Regulates Multilineage Differentiation in Stem Cells From Human Exfoliated Deciduous Teeth
Source: Clin Exp Dent Res. 2025 Nov 20;11(6):e70248. doi: 10.1002/cre2.70248 (PMC12631897; doi:10.1002/cre2.70248)
Supplement: Supplementary file 2 — Supporting Table 1: Oligonucleotide sequence. [file CRE2-11-e70248-s002.docx]

**Supplementary Table 1.** Primer sequences.

**Gene Accession Number Primer sequences**

*COL1A1* NM000088.4 (Forward) 5’ GTGCTAAAGGTGCCAATGGT 3’

(Reverse) 5’ ACCAGGTTCACCGCTGTTAC 3’

*SPP1*  NM001040058.2 (Forward) 5’ AGGAGGAGGCAGAGCACA 3’

(Reverse) 5’ CTGGTATGGCACAGGTGATG 3’

*DSPP* NM014208.3 (Forward) 5’-GCGCAAACGAAAGAGATACCAAC-3’

(Reverse) 5’-CAGGTCGTCGTCACCGTTGTCTTT-3’

*RUNX2* NM001369405.1 (Forward) 5’ ATGATGACACTGCCACCTCTG 3’

(Reverse) 5’ GGCTGGATAGTGCATTCGTG 3’

*LPL*  NM000237.3 (Forward) 5’ GAGATTTCTCTGTATGGCACC 3'

(Reverse) 5' CTGCAAATGAGACACTTTCTC 3'

*PPARG*  NM001354668.2 (Forward) 5′CCAGTGGTTGCAGATTACAAGTATG 3′

(Reverse) 5′ TTGTAGAGCTGAGTCTTCTCAGAATAATAAG 3'

*CEBPA* NM001287424.2 (Forward) 5’-CAACGACAAGAACAGGTGGC-3’

(Reverse) 5’-CGGTCCTGATCCTCTAAGGC-3’

*BGLAP* NM001199662.1 (Forward) 5’ CTTTGTGTCCAAGCAGGAGG 3’

(Reverse) 5’ CTGAAAGCCGATGTGGTCAG 3’

*RANKL*  NM_003701.4 (Forward) 5’-TCAGCCTTTTGCTCATCTCACTAT-3’

(Reverse) 5’-CCAACCCCGATCATGGT-3’

*OPG*  NM_002546.4 (Forward) 5’-AGCTGCAGTACGTCAAGCAGGA-3’

(Reverse) 5’-TTTGCAAACTGTATTTCGCTCTGG-3’

*GAPDH* NM_002046.7 (Forward) 5’-TCATGGGTGTGAACCATGAGAA-3’ (Reverse) 5’-GGCATGGACTGTGGTCATGAG-3’
